# Time trends in public stigma

**DOI:** 10.1192/j.eurpsy.2025.56

**Published:** 2025-08-26

**Authors:** G. Schomerus, Mental Health and Society Working Group, Leipzig University

**Affiliations:** Department of Psychiatry, Leipzig University, Leipzig, Germany

## Abstract

**Abstract:**

Attitudes toward people with mental illness change. We present evidence of attitude change from different contries, with regard to different mental health conditions. While attitudes towads mental health problems in general, and towards depression, seem to soften, there is little positive change with regard to people with substance use disorders, and attitudes have even worsened towards people with schizophrenia. We discuss possible explanations for these diverging trends, and hypothesize on potential remedies.

**Disclosure of Interest:**

None Declared

